# Why Breast Cancer Risk by the Numbers Is Not Enough: Evaluation of a Decision Aid in Multi-Ethnic, Low-Numerate Women

**DOI:** 10.2196/jmir.4028

**Published:** 2015-07-14

**Authors:** Rita Kukafka, Haeseung Yi, Tong Xiao, Parijatham Thomas, Alejandra Aguirre, Cindy Smalletz, Raven David, Katherine Crew

**Affiliations:** ^1^ Columbia University, College of Physicians and Surgeons Biomedical Informatics Mailman School of Public Health, Sociomedical Sciences New York, NY United States; ^2^ Columbia University, Teachers College Health and Behavior Studies New York, NY United States; ^3^ Mailman School of Public Health, Columbia University Epidemiology New York, NY United States; ^4^ MD Anderson Cancer Center Department of Clinical Cancer Prevention Houston, TX United States; ^5^ Columbia University Community Engagement Resource, Irving Institute for Clinical & Translational Research New York, NY United States; ^6^ Columbia University Center for New Media Teaching and Learning New York, NY United States; ^7^ Columbia University Biomedical Informatics New York, NY United States; ^8^ Columbia University, College of Physicians and Surgeons Medicine Herbert Irving Comprehensive Cancer Center New York, NY United States

**Keywords:** breast cancer, decision making, risk communication, consumer health informatics, genetic testing, decision aid, risk stratified screening

## Abstract

**Background:**

Breast cancer risk assessment including genetic testing can be used to classify people into different risk groups with screening and preventive interventions tailored to the needs of each group, yet the implementation of risk-stratified breast cancer prevention in primary care settings is complex.

**Objective:**

To address barriers to breast cancer risk assessment, risk communication, and prevention strategies in primary care settings, we developed a Web-based decision aid, RealRisks, that aims to improve preference-based decision-making for breast cancer prevention, particularly in low-numerate women.

**Methods:**

RealRisks incorporates experience-based dynamic interfaces to communicate risk aimed at reducing inaccurate risk perceptions, with modules on breast cancer risk, genetic testing, and chemoprevention that are tailored. To begin, participants learn about risk by interacting with two games of experience-based risk interfaces, demonstrating average 5-year and lifetime breast cancer risk. We conducted four focus groups in English-speaking women (age ≥18 years), a questionnaire completed before and after interacting with the decision aid, and a semistructured group discussion. We employed a mixed-methods approach to assess accuracy of perceived breast cancer risk and acceptability of RealRisks. The qualitative analysis of the semistructured discussions assessed understanding of risk, risk models, and risk appropriate prevention strategies.

**Results:**

Among 34 participants, mean age was 53.4 years, 62% (21/34) were Hispanic, and 41% (14/34) demonstrated low numeracy. According to the Gail breast cancer risk assessment tool (BCRAT), the mean 5-year and lifetime breast cancer risk were 1.11% (SD 0.77) and 7.46% (SD 2.87), respectively. After interacting with RealRisks, the difference in perceived and estimated breast cancer risk according to BCRAT improved for 5-year risk (*P*=.008). In the qualitative analysis, we identified potential barriers to adopting risk-appropriate breast cancer prevention strategies, including uncertainty about breast cancer risk and risk models, distrust toward the health care system, and perception that risk assessment to pre-screen women for eligibility for genetic testing may be viewed as rationing access to care.

**Conclusions:**

In a multi-ethnic population, we demonstrated a significant improvement in accuracy of perceived breast cancer risk after exposure to RealRisks. However, we identified potential barriers that suggest that accurate risk perceptions will not suffice as the sole basis to support informed decision making and the acceptance of risk-appropriate prevention strategies. Findings will inform the iterative design of the RealRisks decision aid.

## Introduction

Breast cancer confers significant morbidity and mortality on women in the United States, and the primary prevention of this disease is a major public health issue. In 2014, an estimated 232,670 women in the United States will be diagnosed with breast cancer and 40,000 women will die from this disease [1]. Known breast cancer risk factors include age, family history, benign breast disease, reproductive history, and lifestyle factors, such as alcohol intake and obesity [2]. Genetic determinants, such as BRCA1 and BRCA2 mutations, confer the greatest impact on breast cancer risk with a 40-60% lifetime risk [3]. The Gail breast cancer risk assessment tool (BCRAT) is the most commonly used model in the United States and provides an individual’s absolute 5-year and lifetime risk of invasive breast cancer compared to the general population [4]. Breast cancer risk assessment, including genetic testing for hereditary breast cancer, is underutilized in the United States [5]. Many women may be unaware of their risk status due to our inability to adequately screen them in the primary care setting. Using a risk-stratified approach, breast cancer screening and preventive options could be tailored to an individual’s risk profile to maximize benefits and minimize harms. Barriers to adopting risk-appropriate screening and prevention include inaccurate risk perceptions, inadequate time for counseling, insufficient knowledge about risk-reducing strategies, and a number of potential ethical and social issues [5-8].

Women from racial/ethnic minorities are less likely to seek breast cancer preventive care [9,10], contributing to higher rates of late stage diagnosis and poorer clinical outcomes in these populations compared to non-Hispanic whites [11-13]. Low numeracy (ie*,* the effective use of quantitative information to guide health behavior and make health decisions) affects up to 93 million Americans and constrains counseling about cancer risk and prevention strategies [14,15]. We previously reported that low-numerate patients were more likely than high-numerate patients to overestimate their risk [15]. Most research to date that has focused on explaining risks to patients in narratives, numbers, or graphs reveals that all three forms can produce reasoning biases that complicate risk communication [15-17]. People overweigh rare events when they read described probabilities but assign them lower weight when they experience probabilities through an activity such as drawing cards from a deck [18,19]. When participants used an experience-based dynamic interface to interpret risk in our previous study, the differences in risk perceptions associated with low numeracy were reduced [20].

The purpose of this study is to conduct focus groups to inform the iterative design of RealRisks, a patient-centered decision aid for communicating breast cancer risk, reducing inaccurate risk perceptions, and providing preference-based decision support for risk management. When integrated into clinical workflow, RealRisks will identify high-risk women, present them with a unique experience-based dynamic interface to communicate risk, and facilitate communication between patients and clinicians about the risks and benefits of appropriate preventive strategies. We targeted a multi-ethnic population of women from New York City with a high proportion of low numeracy.

## Methods

### Recruitment

In June 2013, we conducted four focus groups among English-speaking women, age ≥18 years, recruited from Northern Manhattan in New York, New York. Women who participated in a community database through the Community Engagement Core Resource of the Irving Institute for Clinical and Translational Research were contacted via email or telephone. Each focus group consisted of 7-9 women. The study was approved by the Institutional Review Board at Columbia University Medical Center, and all participants provided written informed consent. A total of 34 women that reside in the Washington Heights/Inwood community participated.

### Description of the RealRisks Decision Aid

**Figure 1:**
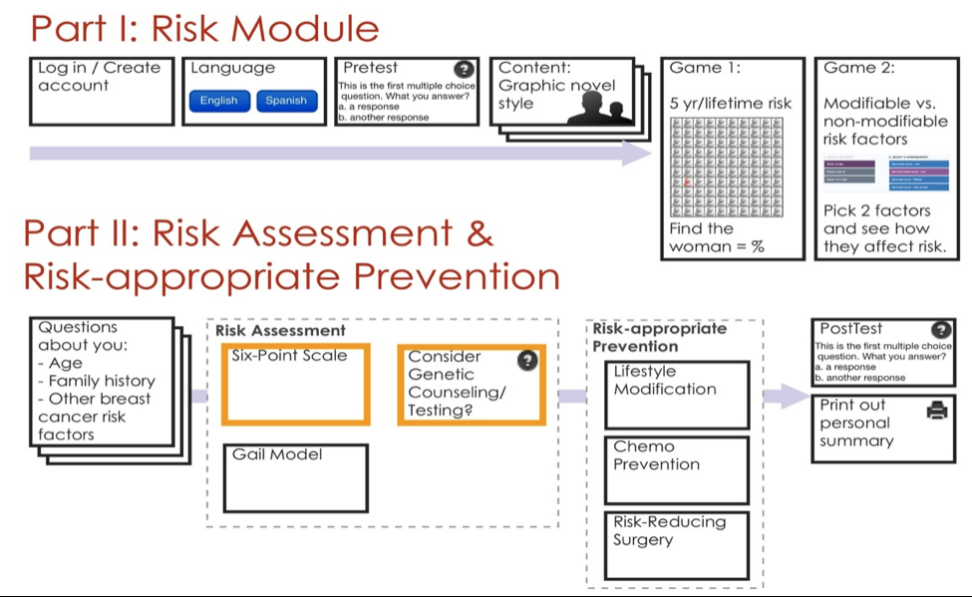
Schema of the RealRisks decision aid.

RealRisks models patient-provider dialogue and incorporates experience-based dynamic interfaces to communicate numeric and probabilistic concepts that are central to risk communication (Figure 1). The narrative is based on a fictitious character Rose, who has a family history of breast cancer and visits her doctor for a routine check-up. We segmented the narrative into the following modules: (1) risk (what is risk, what are breast cancer risk factors), (2) genetic testing (hereditary breast cancer, inherited mutations), and (3) chemoprevention (anti-estrogens, risks/benefits). Embedded within the narrative of RealRisks are two games of experience-based risk interfaces, based upon our previous work [21]. The games demonstrate absolute 5-year and lifetime breast cancer risk for an average 50-year-old woman using a pictograph with 100 clickable women. Players are instructed to click until they “find” a woman with breast cancer. Players continue to click (eg, sample from the population of 100 women) to better learn the meaning of a given pre-set probability (ie*,* 12 out of 100 women or 12%). Our data suggest that this interactive experience-based format for representing risk improves accuracy of risk perception in a low-numerate population [21].

### Conducting the Focus Groups

A skilled facilitator (ANA) led the focus groups using detailed guides (available from the authors upon request). The discussion guide included questions on breast cancer risk factors, BRCA genetic testing, and discussing breast cancer risk and/or genetic testing with a provider. The sessions lasted about 90 minutes and were audio recorded. For the first 15 minutes, women participated in a discussion about their experiences with breast cancer and how they understood breast cancer risk. For the next 30 minutes, the participants were allowed to view and interact with the RealRisks decision aid on a laptop and listen to an audio recording of the narrative. The last 30 minutes involved a semistructured group discussion to obtain feedback on the acceptability and usefulness of the DA. Specifically, we were interested in learning: (1) Do users accept the decision aid for learning about breast cancer risk and genetic testing?, (2) Can users easily navigate and use the decision aid?, (3) Does the decision aid effectively increase users’ confidence and participation in the decision-making process?, and (4) Does the decision aid increase knowledge, understanding of breast cancer risk, and risk management options in the context of an individual’s risk profile?

Prior to starting the focus group discussions, a self-administered questionnaire including information about demographics, numeracy [22], Internet access, sources of information, and breast cancer risk factors was completed at baseline. Perceived breast cancer risk using a validated measure [23] was assessed before and after exposure to RealRisks and evaluation of the decision aid on a 7-point Likert scale was administered post intervention.

### Quantitative Analysis

Descriptive statistics were generated to document participant baseline characteristics and frequencies of positive and negative attitudes about the RealRisks decision aid. Perceived breast cancer risk was assessed by asking “What is your best guess about your percent chance of developing breast cancer during the next 5 years?” and “during your lifetime?” on a scale from 0% to 100% [22]. The Gail BCRAT was used to estimate absolute 5-year and lifetime invasive breast cancer risk [4]. Accuracy of perceived breast cancer risk was defined as within ±5% of estimated lifetime risk according to the BCRAT. Paired *t* test and McNemar’s test were used to compare within-individual changes in accuracy of perceived breast cancer risk before and after interacting with RealRisks.

### Qualitative Analysis

For the qualitative analysis, 2 investigators (HSY and TX) independently read the transcript from the first completed focus group to develop the initial codes and coding template. We identified meaningful segments within the responses and assigned codes using an editing style analysis [24]. Discrepancies in coding were negotiated at weekly research meetings. HSY and TX independently read and coded the remaining three focus group transcripts, applying the coding template, which was iteratively modified as the analysis proceeded. We grouped codes into general themes and discussed the themes among the entire team of investigators. The team collectively selected the themes and representative quotes presented in this paper. Atlas.ti 7.0 software was used to facilitate qualitative data management and analysis. All transcripts were uploaded into the software to enable investigators to do coding, build the codebook, and group the codes into themes. A final comparison of coding across all interviews yielded 62.3%-94.5% agreement. Thematic saturation was determined based on consensus of the coders that new thematic categories were no longer arising from the final focus group transcript.

## Results

### Participant Characteristics

The baseline characteristics of the 34 focus group participants are summarized below (Table 1). The majority (62%, 21/34) were Hispanic, and mean age was 53 years (range 35-75). A total of 41% (14/34) met criteria for low numeracy, defined as a score of 0-5 (range 0-9) [23], and 65% (22/34) demonstrated poor knowledge of breast cancer risk factors, defined as a score of 0-9 (range 0-18). Everyone had access to the Internet, including 89% (30/34) who used the Internet at least weekly. In terms of breast cancer risk factors, 24% (8/34) of women had a first-degree family history of breast cancer and 13% (4/34) had a prior benign breast biopsy. According to the BCRAT (excluding 3 women with a history of breast cancer), mean absolute 5-year and lifetime risk were 1.11% (range 0.2%-4.3%) and 7.46% (range 2.8%-14.6%), respectively, and 10% (3/34) of women met high-risk criteria for breast cancer (≥1.67% 5-year risk).

**Table 1:** Baseline characteristics of focus group participants (N=34), New York City (2013).

Characteristics of focus group participants		
Age (years), mean (SD)		53.4 (10.2)
**Race/ethnicity, n (%)**		
	Non-Hispanic white	2 (5.9)
	Non-Hispanic black	8 (23.5)
	Hispanic	21 (61.8)
	Asian	1 (2.9)
	Other	2 (5.9)
Low numeracy^a^, n (%)		14 (41.2)
Poor knowledge of breast cancer risk factors^b^, n (%)		22 (64.7)
First-degree family history of breast cancer, n (%)		8 (23.5)
Prior benign breast biopsy, n (%)		4 (12.9)
High risk for breast cancer^c^, n (%)		3 (9.7)
5-year breast cancer risk^d^, mean (SD)		1.11 (0.77)
Lifetime breast cancer risk^d^, mean (SD)		7.46 (2.87)

^a^Numeracy score ranges from 0-9. Low numeracy defined as a score of 0-5 [23].

^b^Score of knowledge of breast cancer risk factors ranges from 0-18, with poor knowledge defined as a score of 0-9.

^c^According to the BCRAT, high risk is defined as 5-year invasive breast cancer risk ≥1.67%.

^d^Excluding 3 women with a prior history of breast cancer.

### Quantitative Analysis

Perceived 5-year and lifetime breast cancer risk ranged from 0-100%. After interacting with RealRisks, the difference in perceived and estimated breast cancer risk according to the BCRAT significantly improved for 5-year risk (*P*=.008), but not for lifetime risk (*P*=.20) (Table 2). Accuracy of perceived breast cancer risk improved from 52% to 70% (*P*=.10). Even in the subgroup of women with low numeracy, accurate risk perceptions improved from 46% to 70%. In particular, 80% (4/5) women who overestimated their lifetime breast cancer risk by more than 30% had accurate risk perceptions after exposure to RealRisks.

Participants’ impressions about the RealRisks decision aid on a 7-point Likert scale are shown in Table 3. Over 75% found the decision aid easy to use and felt that it increased their knowledge about breast cancer, genetic testing, and chemoprevention. Over 87% (29/33) would recommend RealRisks to a friend.

**Table 2:** Accuracy of breast cancer risk perceptions before and after interacting with the RealRisks decision aid among focus group participants (N*=*34), New York City (2013).

Breast cancer risk perception		Before RealRisks	After RealRisks	*P* value
Perceived 5-year breast cancer risk (%), mean (SD)		10.4 (22.4)	5.3 (12.1)	.008^a^
Perceived lifetime breast cancer risk (%), mean (SD)		13.1 (26.1)	9.6 (13.7)	.20^a^
**Accurate perceived breast cancer risk** ^b^ **, n (%)**		15 (51.7)	19 (70.4)	.10^c^
	High numeracy	10 (55.6)	12 (70.6)	
	Low numeracy	5 (45.5)	7 (70.0)	

^a^ *P* value based upon paired *t* test.

^b^Accurate perceived breast cancer risk defined as within ±5% of estimated lifetime breast cancer risk according to the BCRAT.

^c^ *P* value based upon McNemar’s test.

**Table 3:** Evaluation of the RealRisks decision aid on a 7-point Likert scale among focus group participants, New York City (2013).

	Frequency, n (%)		
	Disagree (1-2)	Neutral (3-5)	Agree (6-7)
1. RealRisks is useful	1 (3.0)	9 (27.2)	23 (69.7)
2. Most would learn how to use quickly	0 (0)	13 (39.4)	20 (60.6)
3. Easy to use	0 (0)	4 (12.1)	29 (87.9)
4. Increased knowledge of breast cancer	0 (0)	3 (9.1)	30 (90.9)
5. Increased knowledge of genetic testing	0 (0)	6 (18.2)	27 (81.8)
6. Increased knowledge of chemoprevention	0 (0)	8 (24.2)	25 (75.8)
7. Helped to understand breast cancer risk	0 (0)	11 (33.3)	22 (66.7)
8. Helped to understand lifetime breast cancer risk	1 (3.0)	6 (18.2)	26 (78.8)
9. Helped to understand modifiable risk factors	1 (3.0)	11 (33.3)	21 (63.6)
10. I can relate to Rose	4 (12.1)	9 (27.3)	20 (60.6)
11. Will help to discuss genetic testing with doctor	3 (9.1)	7 (21.2)	23 (69.7)
12. Will help to discuss chemoprevention with doctor	4 (12.1)	8 (24.2)	21 (63.6)
13. More confident about decision making about genetic testing	0 (0)	8 (24.2)	25 (75.8)
14. Less worried about getting breast cancer	2 (6.1)	12 (36.4)	19 (57.6)
15. Women have a choice about getting genetic testing	1 (3.0)	4 (12.1)	28 (84.8)
16. Would recommend RealRisks to a friend	0 (0)	4 (12.1)	29 (87.9)

### Qualitative Analysis

#### Overview

In spite of the improvements in accuracy of perceived breast cancer risk after exposure to RealRisks, three major factors emerged as potential barriers to adoption of risk-appropriate breast cancer prevention strategies (Textbox 1): (1) uncertainty about breast cancer risk and risk models, (2) distrust toward the health care system, and (3) perception that risk assessment to pre-screen women for eligibly for genetic testing may be a proxy for rationing access to care.

**Potential barriers to adopting risk-appropriate breast cancer prevention strategies among focus group participants, New York City (2013).**

Uncertainty about breast cancer risk and risk models:

“As far as constants are concerned, if we were going to do scientific research, the only thing constant is change. I think we are all exposed to things that we are not in control of. So, I think it’s optimistic of us to look at our lineage, to look at people in our family who have had it [breast cancer]. But I think because of external effects, it is hard for us to determine what percentage we might be more susceptible to.”

“I have no idea what the percentage is. No one in my family had breast cancer. They had cysts in their breasts. I have no idea. Nobody smokes in my home. But walking around here, I’m exposed to it.”

“You can’t do catch-up. If it’s in you, you are going to get it [breast cancer].”

“I think the risk [for breast cancer] for everybody is 100%...because anyone can get it.”

“I have 50% chance. My mother died from breast cancer and I had a breast surgery. My daughter had a big lump in her chest at 12. My father had prostate cancer. It’s a horrible death. I still hear my mother screaming.”

“I would like to know if I have the gene mutation. So for me to say that being my mom had it [breast cancer] and take a pill and prevent it and not knowing if I have the gene doesn’t make sense to me.”

Distrust toward the health care system:

“No, because we don’t have the knowledge and don’t ask, and the doctor won’t up and give it to you.”

“If you ask your doctor in the clinic, he’s going to look at you how do you know that? With that look. The condescending attitude. Where did you hear about that?”

“I would look for several doctors. I wouldn’t believe in one doctor’s result I swear because they are all human. The way medicine is going today, it’s not as humanistic as it was. They are not talking to you in the face. They are staring at a bloody computer screen. Dentists are the same. The whole way of practicing medicine today is so highly technical. I would have to seek out a second, third, or maybe even a fourth doctor before I make a decision. I don’t base my decision strictly on what the doctor says. They’ve been wrong at times.”

“Unfortunately we all need to be very proactive and never accept no for an answer. Simply because one or two people said no, they are not going to do the [genetic] test...I think you have to keep going and if it’s something deep down in your heart that is someone should get the test, then they should receive that test.”

“Medical establishment has certain guidelines and recently there is some conflict about mammograms. There is a group, it’s like a renegade group and say you don’t need a mammogram every year, you just need it every 3 or 4 years. Don’t listen to them, because they are rebels. These groups also said you don’t need to do it in your 40s, you just need to do it in your 50s or 60s. These groups always pop up.”

Access to care:

“Why would the health insurance pay for the chemoprevention pill but not for the genetic test to see if you have the gene? I would have an issue with my insurance.”

“The [genetic] test that Angelina Jolie took was $3000, so right now how can everybody find out if they can’t afford it...or if they don’t have insurance?”

“My feeling is that the only deterrent [to genetic testing] would be the cost. If it’s just a blood test, not invasive, what could possibly stop me? For me, personally, it would be the $3000. That’s a biggie...it’s the only thing that would be stopping me.”

“The ones with money can get all this testing done. For people that don’t have money, they are out there.”

#### Uncertainty About Breast Cancer Risk and Risk Models

A consistent theme in our analysis was uncertainty among participants that all factors were accounted for in determining their breast cancer risk. The most commonly expressed concern was the potential for the risk assessment tool to miss something that was unknown and outside their control.

Similar to several prior studies on risk communication, participants referred to quantitative risk with many misconceptions. Having a close family member with cancer appeared to influence a participant’s feeling of risk. Women that relayed their experience with a close friend or family member often used the proverbial “50% chance” to express the idea that anyone can get breast cancer.

Participants viewed any risk, even a low risk, with a degree of uncertainty and therefore not acceptable as the basis for their eligibility to get genetic testing. The perceptions among participants was the “test might uncover the unexpected”. According to one participant who stated her breast cancer risk is 2%: “I would still want to know. There’s always that possibility. There is always that chance and I may not be in the high-risk group and that might be the key to finding out if I carry it [genetic mutation]”. These comments highlight the need for educating patients about when genetic test results are informative or uninformative for estimating cancer risk.

#### Distrust Toward the Health Care System

None of the participants recalled discussing breast cancer risk with their physician. Notably, they expressed their own lack of knowledge as a reason for not initiating a discussion and doubted that their physician would engage. Participants expressed a degree of responsibility to seek out the information they needed to inform their decisions and the need to be proactive because providers won’t “give it up”.

The recent debate about mammographic screening guidelines was discussed in the context of suspicion and distrust. Participants speculated about possible ulterior motives, such as the lack of funding for diseases that concerned women and these perceptions led to reduced credibility of the information they received. These passages highlight how source credibility (providers, health care system in general) may extend perceived uncertainty in breast cancer risk assessment tools.

#### Access to Care

In responding to barriers to genetic testing, not having money was discussed as primary. The financial constraints expressed by women led to many concerns that lack of money or insurance would limit their access. Removing the financial barrier, many of the women expressed the desire to receive genetic testing. As stated by one participant: “If it’s free, I’ll go every 6 months”. Stated by another women: “The ones with money can get all this testing done. For people that don’t have money, they are out there”. Only one woman voiced concern that a woman might regret a decision to “cut their breasts off” based upon their genetic testing results, stating that genetic testing is far too soon to be necessary because “some people will panic”. These comments highlight our participants’ view that use of risk assessment to pre-screen women for eligibly for genetic testing may be perceived as service rationing.

## Discussion

### Principal Findings

In this community of largely Hispanic women from Upper Manhattan, we demonstrated a significant improvement in accuracy of breast cancer risk perception after interacting with the experience-based dynamic interfaces to communicate risk embedded in the RealRisks decision aid. This improvement was found in high numeracy women and also in low numeracy women who systematically have been shown to overestimate their breast cancer risk [25,26].

However, consistent with previous research [27,28], our qualitative analysis showed that women do not associate their disease risk with access to health services—for example, those who perceived their risk to be low still want to be tested for breast cancer susceptibility genes. Thus, more accurate risk perceptions may not suffice as the sole basis to support clinical decision making for breast cancer risk management. We identified three major themes as potential barriers to adoption of risk-appropriate breast cancer prevention strategies. These findings have several implications for communicating risk management options based upon breast cancer risk prediction models.

First, there is a great deal of uncertainty regarding breast cancer risk and the utility of the risk prediction models. There may be misconceptions about testing for breast cancer susceptibility genes—for example, as previously shown, some may incorrectly believe that the BRCA test can diagnose cancer [29]. Prior studies suggest that individuals often reject their personal risk estimates. In the context of colon cancer, one study found that of the participants who correctly remembered their personalized risk of getting colon cancer, only half actually accepted it as valid [30]. With respect to breast cancer, approximately 20% of women did not believe that their personalized risk number was accurate [31]. Women in our study expressed a level of uncertainty that all factors used to determine their breast cancer risk were accounted for. They appeared to justify their perceived risk as better or worse based on their understanding of less well-defined breast cancer risk factors that are not included in the BCRAT model. For example, participants who regularly engage in physical activity may have perceived their personal breast cancer risk as low, whereas participants mentioned being exposed to air pollutants in an urban environment leads to a higher than average breast cancer risk. These so called rational adjustments to risk estimates were similarly shown in a study to evaluate the Alzheimer’s disease risk perceptions of individuals who accurately recall their genetics-based Alzheimer disease risk assessment [32]. While not explicitly discussed, the utterances of the women in our focus groups recognized that risk models are imperfect tools. Drawing an analogy to Taleb’s “black swan” logic [33], the inability to predict outliers (black swans) implies the inability to predict those that lie outside the realm of regular expectations. Women frequently gave examples of what we are not in control of and unexpected events (eg*,* the 35-year-old woman diagnosed with breast cancer without clear risk factors).

Prominent in our analysis was the perception that health care itself was the source of distrust, which contributes to the uncertainty of the breast cancer risk prediction models. It is well understood that one of the most important determinants of responses to risk information is likely to be whether the information source is perceived to be credible and trustworthy. Eagley, Wood, and Chaiken have identified two types of communicator bias that message recipients might infer [34]. The first is knowledge bias, which refers to a belief that the communicator’s knowledge about truth is adequate. In the context of risk assessment models, this might refer to the perception that the tool itself can calculate probabilities to express uncertainty (eg, are the important factors considered and is the science about known risk factors adequate?). The second is reporting bias. In the context of our study, this might refer to a belief that the provider and health care system more generally is not distorting information in order to promote a particular view. Our participant’s statement that it is the “renegade group” saying you no longer need a mammogram every year expresses the concern that recommendations about screening mammography are distorted and should not be trusted. While our study was not designed to explore the mechanisms contributing to sources of distrust and how such perceptions might influence risk models, our findings build on prior work suggesting that cancer risk assessment models can be viewed with uncertainty [35,36] and distrust [27,30,31] and that source credibility is a key determinant [35,36].

Another novel finding was the perception of using risk models to guide breast cancer risk management options as a barrier to access to care. Some of the women in our focus groups viewed not being eligible for genetic testing as service rationing. As one woman stated “only the ones with money can get all this testing done”. The well-known Angelina Jolie effect resulted in demand for BRCA testing to almost double [37]. Although a survey carried out in the United States found that although 75% of Americans were aware of Angelina Jolie’s double mastectomy, fewer than 10% of respondents had the information necessary to accurately interpret her risk of developing cancer relative to a woman unaffected by the BRCA gene mutation [38]. Awareness of the Angelina Jolie story was not associated with improved understanding. The women in our study estimated the cost of genetic testing to be about US $3000, which raised the question “right now, how can everybody find out if they can’t afford it?”. In order to avoid undermining wider trust in the health care system, effective communication strategies are needed to ensure that those designated as low-risk understand and trust that the rationale behind tailoring prevention regimens is not about reducing or withholding services, but rather it is to optimize benefits and reduce potential harms. The complexities of this communication could be most problematic for women with low numeracy, as they tend to overestimate their risk. Therefore, active communication and assurances are critical to mitigating any exacerbation of existing disparities.

While underutilized in the United States, breast cancer risk assessment including genetic testing can be used to classify people into different risk groups with screening and preventive interventions tailored to the needs of each group [5]. By risk-stratifying the population, screening and management options could be applied differentially to each population stratum with potentially more efficient allocation of resources. However, even if improved health outcomes are achieved, the implementation of risk-stratified breast cancer prevention programs has proven to be complex [39-41]. Currently, there is a scarcity of empirical evidence about patient acceptance and their preferences with regard to risk-appropriate cancer screening and prevention [41].

### Limitations

Our urban-dwelling female participants, who were largely minorities with a high proportion of low numeracy, may not be representative of the general population in other geographic areas. However, given that this is an understudied and underserved population, this is also a unique strength of our study. There may have also been selection bias, where women with a greater interest in expressing their opinions about breast cancer may have been more likely to participate, which affects the generalizability of our results.

### Conclusions

**Figure 2:**
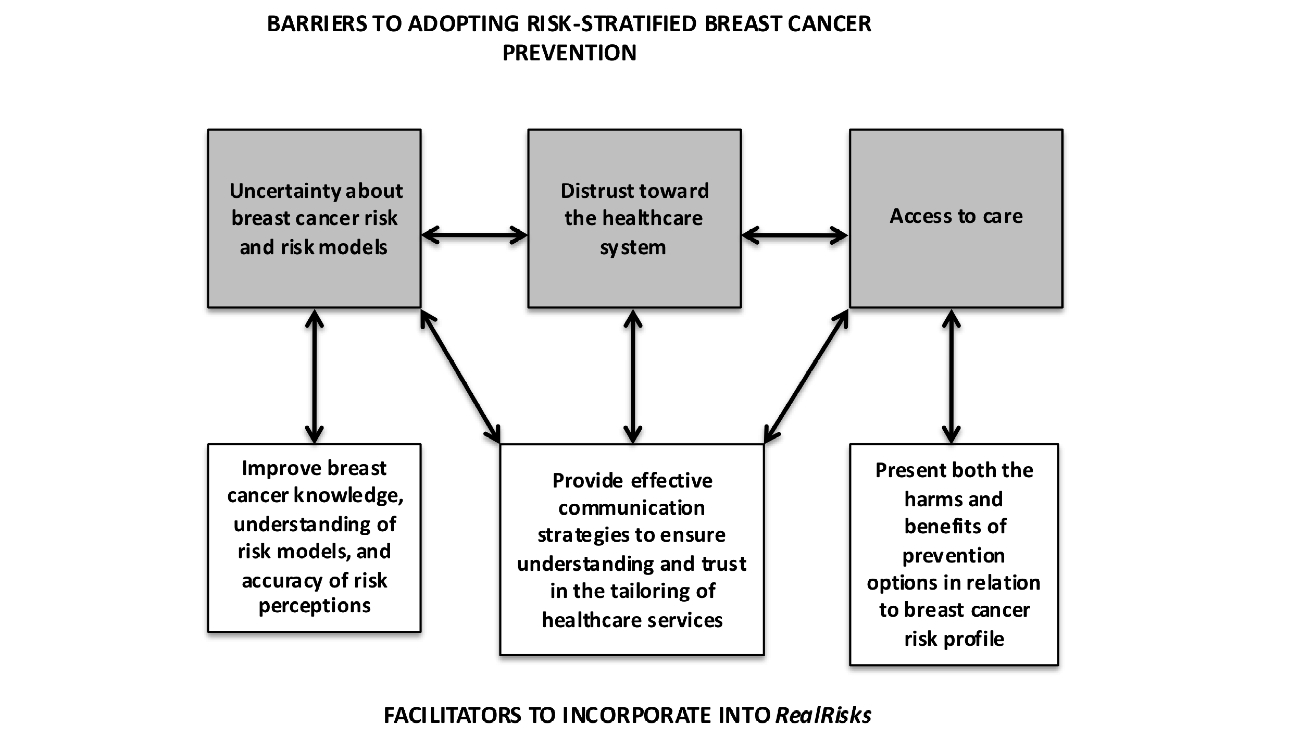
Schema of barriers and facilitators to the adoption of breast cancer risk assessment and risk-appropriate prevention strategies, which will inform the iterative design and refinement of the RealRisks decision aid.

We conducted this study to inform the iterative design of the RealRisks decision aid. While the experience-based interface resulted in improved accuracy of breast cancer risk perceptions, our qualitative findings identified additional barriers to risk-based health care delivery, which need to be addressed (Figure 2). For example, to address the theme of distrust toward the health care system, RealRisks will now incorporate dialogue to explain that genetic testing has few or no benefits for women who do not have a family history that is associated with increased risk for BRCA1 or BRCA2 mutations. The experience-based interface will be extended to include how taking chemoprevention pills might impact their personalized risk, so they can learn by interacting with the game that benefit is seen only among high-risk women and risks outweigh benefits for women below a specified risk threshold. Moreover, we will emphasize using both dialogue and games that women across all risk strata have preventive options. The game will allow women to learn about screening and lifestyle choices. We consider this to be particularly salient as controversy over the potential harms of population-based mammographic screening due to overdiagnosis continues to escalate [42,43]. Future studies are needed to determine how these iterations to RealRisks are received, and more generally, whether decision aids, such as RealRisks, can improve accuracy of breast cancer risk perceptions, informed decision making, and acceptance of risk-appropriate prevention strategies.

## Abbreviations

BCRAT: breast cancer risk assessment tool
BRCA: BReast CAncer
